# Durational Evidence That Tokyo Japanese Vowel Devoicing Is Not Gradient Reduction

**DOI:** 10.3389/fpsyg.2019.00821

**Published:** 2019-04-16

**Authors:** James Tanner, Morgan Sonderegger, Francisco Torreira

**Affiliations:** Department of Linguistics, McGill University, Montreal, QC, Canada

**Keywords:** phonetics, syllable duration, Japanese, spontaneous speech, vowel devoicing

## Abstract

A central question in the Japanese high vowel devoicing literature concerns whether vowels are devoiced through a categorical process or via gradient reduction. Examining how vowel height and consonantal voicing condition phrase-internal CV duration in a corpus of spontaneous Tokyo Japanese, it was found that CVs containing high vowels are substantially shorter before voiceless consonants, whilst non-high vowels do not exhibit comparable shortening. This quantitative difference between CV durations suggests a controlled temporal compression of the CV, consistent with views that Japanese vowel devoicing is produced through a categorical process targeting high vowels preceding voiceless consonants, and supports previous observations made of elicited productions.

Vowel devoicing is a phenomenon observed in a number of languages (Gordon, 1998), such as Turkish (Jannedy, 1995), French (Torreira and Ernestus, 2010), and Korean (Jun and Beckman, 1993). In Tokyo Japanese, descriptions state that high vowels /i/ and /u/ are near-obligatorily devoiced between two voiceless consonants or following a voiceless consonant pre-pausally (Maekawa and Kikuchi, 2005; Fujimoto, 2015). The former context (between two voiceless consonants) is traditionally described as the “canonical” or “standard” environment for vowel devoicing (Labrune, 2012; Fujimoto, 2015), and is the focus of this paper. A primary question in the Japanese vowel devoicing literature is whether vowels are devoiced via a categorical process (a.k.a. “phonological rule”) or via a gradient reduction process due temporal compression of the laryngeal gestures in articulation (Jun and Beckman, 1993; Fujimoto, 2015). Moreover, substantial debate has considered whether the vowel (i.e., the vocalic target) is deleted versus produced without phonation (e.g., Tsuchida, 1997; Maekawa and Kikuchi, 2005; Nielsen, 2014). The distinction between devoicing and deletion is not of the focus of this paper however, and the term “vowel devoicing” will be used to refer to this phenomenon, regardless as to whether the vowel is deleted or not. Much previous work on vowel devoicing has focused on measuring the “rate” or likelihood of devoicing a particular vowel, often on perceptual evidence (e.g., Fujimoto, 2004; Maekawa and Kikuchi, 2005; Kilbourn-Ceron and Sonderegger, 2018). Instead of devoicing rate, this study focuses on consonant-vowel (CV) syllable duration: a phonetic exponent expected to correlate with the presence of devoicing (Jannedy, 1995)^1^. In this sense, this study is analogous to previous analyses of vowel elision in English (Davidson, 2006) and French (Torreira and Ernestus, 2011) that examine syllable duration as a diagnostic for the nature of reduction processes in connected speech. Due to a lack of clear acoustic landmarks determining where a devoiced vowel “begins” and less prominent formats in devoiced vowels, CV duration in these segmental contexts allows for more consistent segmentation than the vowel itself.

As high vowels are intrinsically shorter than non-high vowels (Solé and Ohala, 2010), and vowels are shorter before voiceless consonants than their voiced counterparts (Chen, 1970), it is possible that high vowels, which are less conducive to voicing when surrounded by obstruents than other vowels, may be sufficiently shortened as to be devoiced (or even acoustically deleted) when they precede a voiceless consonant. Articulatorily, this may be due to the overlap of laryngeal gestures or the inability to reach the appropriate transglottal pressure differential required for voicing (Torreira and Ernestus, 2010). In other words, the main acoustic cues of high vowels are vulnerable (due to their intrinsic shortness and aerodynamic characteristics) when followed by voiceless consonants, particularly under severe temporal constraints. Under a gradient reduction account, high vowel devoicing is produced as a consequence of a more general phonologized shortening process: as opposed to high vowels being specifically targeted, their default shortness (relative to non-high vowels) makes the gestural and aerodynamic conditions untenable for voicing. This account predicts, then, that shortening before voiceless consonants should be observed for *both* CVs containing high and non-high vowels, and the degree of shortening should be comparable between vowel heights. On the other hand, if Tokyo Japanese vowel devoicing is driven by a categorical process that targets vowels in the canonical environment, an asymmetry should be observed between vowel heights: CVs containing high vowels should be substantially shortened before voiceless consonants (e.g., [kit]a vs [kid]a), whilst CVs containing non-high vowels should *not* exhibit comparable shortening (e.g., [kat]a vs. [kad]a). In other words, it is the specific shortening of high vowels in the canonical position that is phonologized for speakers. Thus, the relative degree to which CVs containing high and non-high vowels shorten before voiceless consonants could be used as evidence of the mechanisms behind vowel devoicing.

Examining how CV duration is modulated is also related to previous research on prosodic timing in Japanese, where it has been suggested that Japanese compensates for phonological and phonetic effects on duration in order to maintain similar durations across moras (Port et al., 1987). It is not clear whether this form of compensation is maintained in spontaneous speech, however, and it has been claimed Japanese timing is derived from other phonological factors (Warner and Arai, 2001). With respect to vowel devoicing, this kind of durational compensation should predict that the preceding consonant should lengthen to account for the loss of vowel duration (Han, 1994), which in turn would nullify a prospective CV duration effect from vowel devoicing. This study aims to compare the categorical process and gradient reduction accounts of vowel devoicing behavior by examining how the effect of following consonant voicing on CV duration is modulated by vowel height in a corpus of spontaneous Japanese speech. As previous studies have examined the modulation of vowel duration in single-word utterances (which constitute their own prosodic unit) or scripted carrier phrases, this study provides crucial new information about how vowel devoicing is realized in naturalistic connected speech.

## 1. Methods

The data come from 317,707 voiceless-consonant vowel sequences containing high (/i/, /u/) or non-high (/a/, /e/, /o/) vowels extracted from the *Corpus of Spontaneous Japanese* (Maekawa et al., 2000). Whilst this corpus contains data from speakers from different regions in Japan, the majority of speakers (131 of 137) are classed as being from Tokyo or another city in the Greater Tokyo Metropolitan Area. Furthermore, the speech in this corpus is of a variety referred to as “Common Japanese”: a variety used in business and professional situations which draws much of its phonological, syntactic, and lexical properties from the Tokyo dialect (Maekawa et al., 2002). To compare the effect of following consonant voicing, only CVs that were followed by either stops, affricates, or fricatives were retained, resulting in 196,130 exclusions. As high pitch accents and boundary tones block the application of devoicing (Fujimoto, 2015), 96,716 CVs containing either an accent or high boundary tone were also excluded. Phrasal position was defined using the X-JToBI system (Maekawa et al., 2002), a desciptive mechanism of defining Japanese prosodic structure based on the presence of tones, which was manually annotated in this corpus (Kikuchi and Maekawa, 2003). In this study, CVs preceding Break Indices {0,1} were considered “phrase-internal,” and {2,3} for “phrase-final” CVs. As phrase-final CVs often co-occur with the presence of pauses (22% in this dataset), boundary tones, and segmental lengthening (Ueyama, 1999), only phrase-internal CVs were included for the analysis, resulting in the exclusion of 16,517 phrase-final CVs. Additionally, the focus on phrase-medial contexts provides a better comparison to previous research on the articulatory mechanisms of Japanese vowel devoicing in the canonical environment, which have predominantly focused on word-internal devoicing (e.g., Jun and Beckman, 1993; Fujimoto and Kiritani, 2003; Fujimoto, 2004). In total, 80,189 tokens (43,173 high; 37,016 non-high) were used in the analysis corresponded to 4,789 unique words, spoken by 137 speakers (58 female). CV duration was calculated as the difference between the start and end times of the CV, as defined by the hand-corrected annotations provided with the corpus (Kikuchi and Maekawa, 2003). Speech rate was calculated as the phones per second within a single inter-pausal unit, from which a mean value was calculated for each speaker (which can thus be interpreted as faster vs. slower speakers), and a “local” rate (calculated as raw−mean), which can be interpreted as (faster vs. slower speech for that speaker).

**Figure 1:**
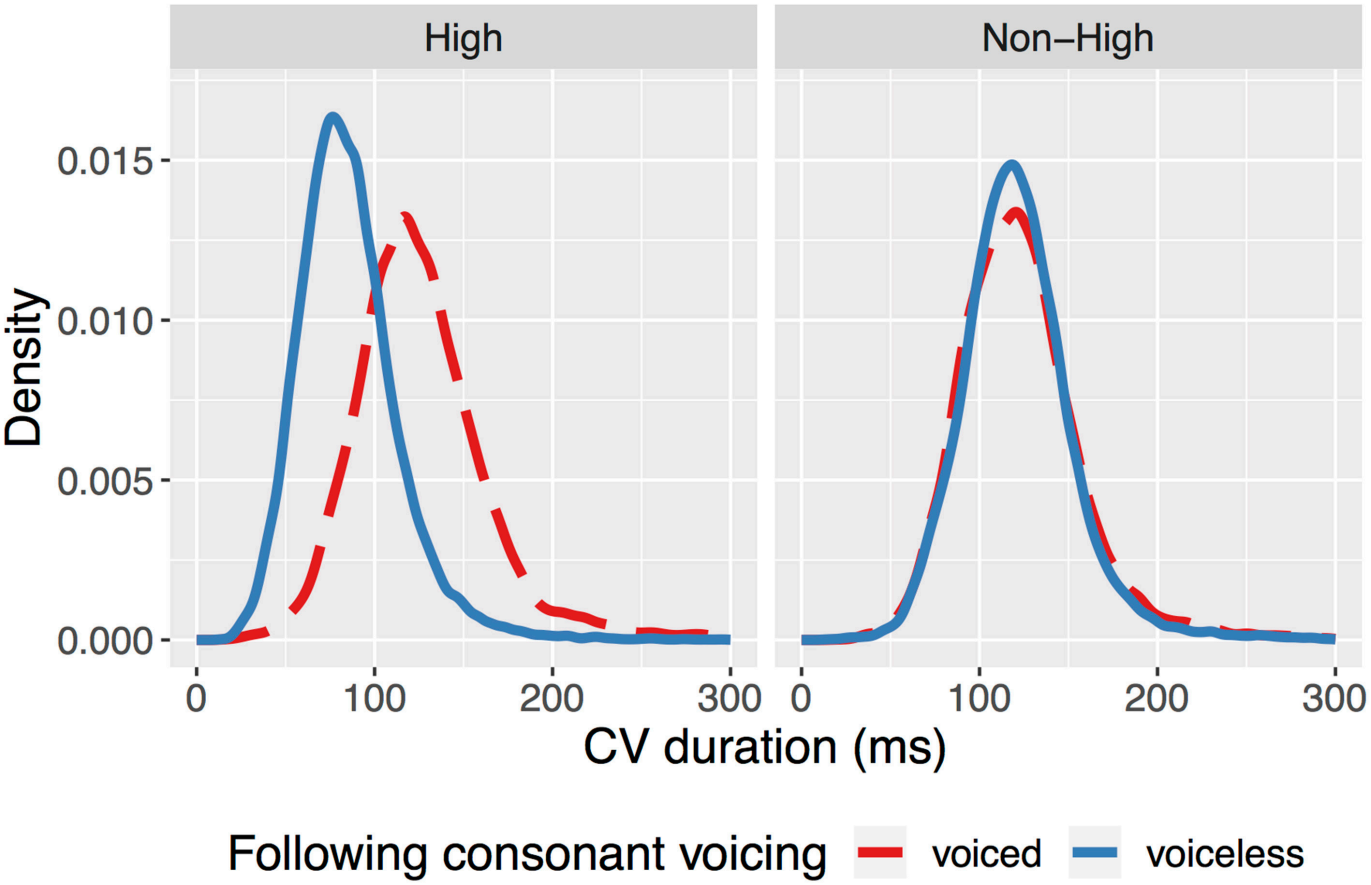
Kernel density of CV duration containing a voiceless consonant and a high vowel **(Left)** or a non-high vowel **(Right)**.

A mixed-effects linear regression model was fit to CV duration using the lmerTest package (Kuznetsova et al., 2017) in *R* (R Core Team, 2017). To examine the variables of interest, the fixed-effects structure contained predictors for following consonant voicing, vowel height, and an interaction between them. These interaction terms model the different configurations of vowel height and following consonant voicing as seen in the kernel density plot in Figure 1^2^, where following voicing is compared for each level of vowel height. As Tokyo Japanese vowel devoicing is also known to be conditioned by speech rate, lexical frequency, and the manner of the surrounding consonants (Kilbourn-Ceron and Sonderegger, 2018), these factors were also included as controls in the model. Two-level predictors (i.e., voicing, height) were converted into numerical predictors (with range 1) and centered. Continuous predictors were centered and divided by two standard deviations. The three-level predictors of preceding and following consonant manner were sum-coded, with “stop” as the reference level. The model was fit with full possible by-word and by-speaker random intercepts and slopes that would enable model convergence, with correlations between random effects omitted (Barr et al., 2013)^3^.

## 2. Results

The full model table for all predictors can be seen in Table 1. The control variables influenced CV duration in the expected directions based on previous work modelling a perceptual measure of devoicing (Kilbourn-Ceron and Sonderegger, 2018). For ease of interpretation, results are reported on the degree of shortening as differences in medians ($\Delta \tilde{x}$) and pairwise comparisons of estimated marginal means (averaging over categorical variables and holding continuous variables at their mean values) between voiced and voiceless consonants at each vowel height ($\Delta \hat{\beta }$), computed using *emmeans* (Lenth, 2018). As shown in the distribution of CV durations in Figure 1, high-vowel-CVs are 25% shorter before voiceless consonants compared voiced consonants ($\Delta \tilde{x}$ = −39.32; $\Delta \hat{\beta }$ = −31.34, *p* < 0.001), whilst non-high-vowel-CVs shorten by 3% ($\Delta \tilde{x}$ = −0.61; $\Delta \hat{\beta }$ = −4.22, *p* = < 0.005), and the difference between the degree of shortening in both environments is significant ($\Delta \hat{\beta }$ = −27.12, *p* < 0.001). Whilst shortening occurs across both vowel heights, the degree of shortening in non-high contexts is substantially less than that reported for languages in Chen (1970), and is consistent with the view that Japanese maintains some durational equivalence of CV units before voiced and voiceless consonants (Shaw and Kawahara, 2017). The fact that this temporal similarity is not maintained across consonantal contexts for high vowels suggests that only CVs containing high vowels are distinctly shortened in this environment. It should be noted, however, that the tokens used in Chen (1970) contained the vowel and consonant within the same syllable: given that this is not the case for the environments examined here (where the following consonant is a part of the following syllable), this raises a broader question about how the vowel shortening effect is cross-linguistically modulated by whether the consonant appears either in the same or following syllable to that of the vowel.

**Table 1:** Fixed effect coefficients ($\hat{\beta }$), standard errors (SE($\hat{\beta }$)), degrees of freedom, *t*-scores, and *p*-values (calculated using Satterthwaite approximation) for all model predictors.

**Predictor**	**$\hat{\beta }$**	**SE($\hat{\beta }$)**	**df**	**t**	**Pr(>**|**t**|**)**
Intercept	122.128	1.180	428.313	103.459	<0.001
Consonant voicing	17.804	0.996	261.223	17.881	<0.001
Vowel height	31.920	0.993	357.136	32.140	<0.001
Speech rate (mean)	−23.650	1.580	145.163	−14.971	<0.001
Speech rate (local)	−20.624	0.718	590.708	−28.708	<0.001
Frequency	−2.129	0.650	1, 358.434	−3.273	0.001
**NEXT PHONEME MANNER**					
*Affricate*	−3.633	0.755	1, 442.006	−4.814	<0.001
*Fricative*	4.120	1.209	748.417	3.408	<0.001
**PREVIOUS PHONEME MANNER**					
*Affricate*	−5.469	0.817	1, 186.380	−6.690	<0.001
*Fricative*	13.287	1.185	1, 084.729	11.211	<0.001
Voicing : Height	−27.119	1.356	903.131	−20.003	<0.001
**NEXT MANNER : PREVIOUS MANNER**					
*Affricate : Affricate*	0.252	0.799	1, 380.341	0.316	0.752
*Fricative : Affricate*	−2.455	1.349	839.688	−1.819	0.069
*Affricate : Fricative*	−1.760	1.233	1, 385.133	−1.428	0.154
*Fricative : Fricative*	11.699	2.127	935.983	5.502	<0.001

## 3. Discussion

The aim of this study was to investigate the underlying articulatory mechanisms of vowel devoicing in Tokyo Japanese by examining how phrase-internal CV duration is modulated by the relationship between vowel height and following consonantal voicing. If high vowel devoicing was caused by a general shortening of CVs before voiceless consonants, it would have been expected that all CVs (regardless of the height of the vowel) would be shorter before a voiceless consonant, and that the degree of shortening would be similar across both vowel heights. The reason why only high vowels would undergo devoicing in this scenario would be due to the additive effect of a general shortening process on top of the inherent shortness of high vowels, causing the overlap of laryngeal gestures and/or the inability to maintain the necessary transglottal pressure differential required for voicing. What is observed in Figure 1, however, is a substantial temporal compression of high-vowel CVs before voiceless consonants without equivalent shortening in CVs containing non-high vowels. This qualitatively different behavior of high vowels suggests that Japanese vowel devoicing is not the consequence of a generalized shortening mechanism driven by the voicelessness of the following consonant, but instead is consistent with the view that vowel devoicing is a targeted, controlled process that exclusively affects high vowels in a specific phonological context (Fujimoto et al., 2002; Nielsen, 2015): namely, between voiceless obstruents with no associated high boundary tone or lexical pitch accent (Fujimoto, 2015; Kilbourn-Ceron and Sonderegger, 2018). Fujimoto et al. (2002) suggest that devoicing in these cases is produced by a reorganization of glottal gestures, where the closing of the glottis to produce voicing is simply bypassed. In this study, however, CVs are also significantly shorter in their supraglottal articulations. This suggests that Japanese devoicing is not simply a phenomenon concerning glottal coordination, but also involves controlled temporal reduction at the supraglottal level (see Fujimoto and Kiritani (2003) for a similar conclusion regarding laboratory speech). Observing this result in spontaneous connected speech further supports the view that controlled temporal modulation is utilized in producing canonically-devoiced vowels in Japanese. With respect to the compensation of mora length, the results of this study suggest that a strong version of the compensation hypothesis (that the preceding consonant lengthens to account for vowel shortening) does not straightforwardly apply to cases of vowel devoicing in spontaneous speech (contra Han, 1994). Whilst it is possible for speakers to compensate for the consequences of some phonological processes, it is apparent that this is not true for vowel devoicing^4^.

## 4. Conclusion

This study examined how the CV duration is modulated as a function of vowel height and consonantal voicing in a corpus of Tokyo Japanese spontaneous speech, as a means of investigating the underlying mechanisms involved in Japanese vowel devoicing. The quantitative difference observed between high and non-high-vowel CVs can be interpreted as support for the view that high vowels are targeted as part of a controlled devoicing process involving substantial temporal compression, as opposed to a general reduction process gradiently applying to all vowels before voiceless consonants. As the findings of this study are based exclusively on acoustic evidence, however, further articulatory studies [e.g., Shaw and Kawahara (2018)] are needed. By utilizing spontaneous speech, however, this study has supported and expanded on previous laboratory research, providing further insight into the underlying mechanisms of Japanese high vowel devoicing.

## Author Contributions

JT extracted the data, performed the statistical analysis, and wrote the first draft of the manuscript. All authors contributed conception and design of the study. All authors contributed to manuscript revision, read, and approved the submitted version.

### Conflict of Interest Statement

The authors declare that the research was conducted in the absence of any commercial or financial relationships that could be construed as a potential conflict of interest.

## Footnotes

^1^The status of syllables as a phonological unit in Japanese has been debated (Labrune, 2012), though there is strong evidence that Japanese utilizes syllabic units for the purposes of applying phonological rules (Kawahara, 2016). This debate is not crucial for this study, however, as only CV units are examined (as opposed to syllables containing moraic nasals or geminates).

^2^Kernal density plots illustrate the distribution of the data along a continuous variable (in this case duration) without requiring the data to be subset into pre-defined bins, as is the case with histograms.

^3^The linear model was specified as (in R syntax): lmer(duration ~ voicing * height + speech rate (mean) + speech rate (local) + frequency + previous phoneme manner * following phoneme manner + (1 + voicing * height + previous phoneme manner * following phoneme manner + speech rate (local) + frequency || speaker) + (1 + voicing * height + speech rate (mean) + speech rate (local) + next phoneme manner || word).

^4^Is it not obvious how this related to recent research on mora compensation using this same corpus as this study (Kawahara, 2017), as the effect of vowel devoicing is not addressed.
